# Supplementary material for: Flexible read-aware genotype imputation from sequence using biobank sized reference panels
Source: Nat Commun. 2025 Dec 13;17:524. doi: 10.1038/s41467-025-67218-1 (PMC12804713; doi:10.1038/s41467-025-67218-1)
Supplement: Supplementary file 2 — Reporting Summary [file 41467_2025_67218_MOESM2_ESM.pdf]

nature portfolio

Corresponding author(s): Zilong Li, Robert William Davies

Last updated by author(s): Oct 7, 2025

Reporting Summary

Nature Portfolio wishes to improve the reproducibility of the work that we publish. This form provides structure for consistency and transparency in reporting. For further information on Nature Portfolio policies, see our Editorial Policies and the Editorial Policy Checklist.

Statistics

For all statistical analyses, confirm that the following items are present in the figure legend, table legend, main text, or Methods section.

n/a	Confirmed	
☐	☒	The exact sample size (*n*) for each experimental group/condition, given as a discrete number and unit of measurement
☒	☐	A statement on whether measurements were taken from distinct samples or whether the same sample was measured repeatedly
☐	☒	The statistical test(s) used AND whether they are one- or two-sided<br>*Only common tests should be described solely by name; describe more complex techniques in the Methods section.*
☐	☒	A description of all covariates tested
☐	☒	A description of any assumptions or corrections, such as tests of normality and adjustment for multiple comparisons
☐	☒	A full description of the statistical parameters including central tendency (e.g. means) or other basic estimates (e.g. regression coefficient) AND variation (e.g. standard deviation) or associated estimates of uncertainty (e.g. confidence intervals)
☐	☒	For null hypothesis testing, the test statistic (e.g. *F*, *t*, *r*) with confidence intervals, effect sizes, degrees of freedom and *P* value noted<br>*Give P values as exact values whenever suitable.*
☐	☒	For Bayesian analysis, information on the choice of priors and Markov chain Monte Carlo settings
☒	☐	For hierarchical and complex designs, identification of the appropriate level for tests and full reporting of outcomes
☐	☒	Estimates of effect sizes (e.g. Cohen's *d*, Pearson's *r*), indicating how they were calculated

Our web collection on statistics for biologists contains articles on many of the points above.

Software and code

Policy information about availability of computer code

Data collection	Software was not used for data collection for this study
Data analysis	New code and methods<br>https://github.com/rwdavies/QUILT (main software repository)<br>https://github.com/rwdavies/mspbwt (dependency)<br><br>Custom analysis code:<br>Snakemake imputation workflow (https://github.com/Zilong-Li/lcWGS-imputation-workflow)<br>Scripts for analyses both on the RAP and local machine (https://github.com/Zilong-Li/Papers/tree/main/quilt2)<br><br>Other software used<br>GLIMPSE2 v2.0.0<br>bcftools v1.18<br>samtools v1.18<br>PCAone v0.4.4<br>GEMMA v0.98.5<br>vcfppR v4.6.0<br>shapeit v4.2.2<br>R v4.2.2

For manuscripts utilizing custom algorithms or software that are central to the research but not yet described in published literature, software must be made available to editors and reviewers. We strongly encourage code deposition in a community repository (e.g. GitHub). See the Nature Portfolio guidelines for submitting code & software for further information.

Data

Policy information about availability of data

All manuscripts must include a data availability statement. This statement should provide the following information, where applicable:

- Accession codes, unique identifiers, or web links for publicly available datasets
- A description of any restrictions on data availability
- For clinical datasets or third party data, please ensure that the statement adheres to our policy

This research has been conducted using the UK Biobank Resource under Application No. 32683.

The 1,000 Genomes Project phase 3 dataset sequenced at high coverage by the New York Genome Center is available on the European Nucleotide Archive under accession no. PRJEB31736, the International Genome Sample Resource (IGSR) data portal and the University of Michigan school of public health ftp site (ftp://share.sph.umich.edu/1000g-high-coverage/freeze9/phased/).

The publicly available HRC reference panel is available from the European Genome-phenome Archive at the European Bioinformatics Institute under accession no. EGAS00001001710.

The UKB-200K panel, the UKB-GEL panel, and individuals' WGS data can be accessed via the UKB RAP (https://ukbiobank.dnanexus.com/landing).

The ancient DNA from Afanasievo culture can be accessed via European Nucleotide Archive, accession number PRJEB43093, and the phased VCF file for the family are available from the European Variation Archive, accession number PRJEB46983.

The real NIPT cfDNA data is freely available from the Personal Genome Project: https://my.pgp-hms.org/profile/hu058D3E, and the high coverage sequencing data from saliva of the pregnant mother is available from https://my.pgp-hms.org/profile/huC1F919.

The simulated NIPT samples were simulated as described in the Methods section, and code to perform this simulation is available at https://doi.org/10.5281/zenodo.17316024 .

Research involving human participants, their data, or biological material

Policy information about studies with human participants or human data. See also policy information about sex, gender (identity/presentation), and sexual orientation and race, ethnicity and racism.

Reporting on sex and gender	No sex or gender based analyses were conducted. Biological sex of participants is only used as covariate (provided by the UK Biobank)
Reporting on race, ethnicity, or other socially relevant groupings	N/A (we haven't performed such analyses. we rely on external public source to obtain the information if needed)
Population characteristics	We identify populations using self reported and kinship estimates (provided by the UK Biobank)
Recruitment	N/A (performed by the UK Biobank)
Ethics oversight	N/A (performed by the UK Biobank)

Note that full information on the approval of the study protocol must also be provided in the manuscript.

Field-specific reporting

Please select the one below that is the best fit for your research. If you are not sure, read the appropriate sections before making your selection.

[x] Life sciences [ ] Behavioural & social sciences [ ] Ecological, evolutionary & environmental sciences

For a reference copy of the document with all sections, see nature.com/documents/nr-reporting-summary-flat.pdf

Life sciences study design

All studies must disclose on these points even when the disclosure is negative.

Sample size	Sample size were clearly indicated in the manuscript for each analysis.
Data exclusions	No data were excluded.
Replication	All the software and code used in this study are publicly available as described in the data section.
Randomization	Randomization was not used.
Blinding	Blinding is no relevant to this study.

Reporting for specific materials, systems and methods

We require information from authors about some types of materials, experimental systems and methods used in many studies. Here, indicate whether each material, system or method listed is relevant to your study. If you are not sure if a list item applies to your research, read the appropriate section before selecting a response.

Materials & experimental systems

n/a	Involved in the study
☒	☐ Antibodies
☒	☐ Eukaryotic cell lines
☒	☐ Palaeontology and archaeology
☒	☐ Animals and other organisms
☒	☐ Clinical data
☒	☐ Dual use research of concern
☒	☐ Plants

Methods

n/a	Involved in the study
☒	☐ ChIP-seq
☒	☐ Flow cytometry
☒	☐ MRI-based neuroimaging

Plants

Seed stocks	N/A
Novel plant genotypes	N/A
Authentication	N/A
